# Comparison of Unruptured Intracranial Aneurysm Treatment Score and PHASES Score in Subarachnoid Hemorrhage Patients With Multiple Intracranial Aneurysms

**DOI:** 10.3389/fneur.2021.616497

**Published:** 2021-04-07

**Authors:** Axel Neulen, Tobias Pantel, Jochem König, Marc A. Brockmann, Florian Ringel, Sven R. Kantelhardt

**Affiliations:** ^1^Department of Neurosurgery, University Medical Center of the Johannes Gutenberg-University of Mainz, Mainz, Germany; ^2^Institute of Medical Biostatistics, Epidemiology and Informatics, University Medical Center of the Johannes Gutenberg-University of Mainz, Mainz, Germany; ^3^Department of Neuroradiology, University Medical Center of the Johannes Gutenberg-University of Mainz, Mainz, Germany

**Keywords:** unruptured intracranial aneurysm, ruptured intracranial aneurysm, unruptured intracranial aneurysm treatment score, subarachnoid hemorrhage, PHASES score

## Abstract

**Objective:** Unruptured Intracranial Aneurysm (UIA) Treatment Score (UIATS) and PHASES score are used to inform treatment decision making for UIAs (treatment or observation). We assessed the ability of the scoring systems to discriminate between ruptured aneurysms and UIAs in a subarachnoid hemorrhage (SAH) cohort with multiple aneurysms.

**Methods:** We retrospectively applied PHASES and UIATS scoring to the aneurysms of 40 consecutive patients with SAH and multiple intracranial aneurysms.

**Results:** PHASES score discriminated better between ruptured aneurysms and UIAs than UIATS. PHASES scores and the difference between the UIATS subscores were higher for ruptured aneurysms compared with UIAs, which reached significance for the PHASES score. PHASES score estimated a low 5-year rupture risk in a larger proportion of the UIAs (≤0.7% in 62.3%, ≤1.7% in 98.4%) than of the ruptured aneurysms (≤0.7% in 22.5%, ≤1.7% in 82.5%). In the 40 ruptured aneurysms, UIATS provided recommendation for treatment in 11 (27.5%), conservative management in 14 (35.0%), and was inconclusive in 15 cases (37.5%). In the 61 UIAs, UIATS recommended treatment in 16 (26.2%), conservative management in 29 (47.5%), and was inconclusive in 16 (26.2%) cases.

**Conclusion:** Similar to previous SAH cohorts, a significant proportion of the ruptured aneurysms exhibited a low-rupture risk. Nevertheless, PHASES score discriminated between ruptured aneurysms and UIAs in our cohort; the lower discriminatory power of UIATS was due to high weights of aneurysm-independent factors. We recommend careful integration of the scores for individual decision making. Large-scale prospective trials are required to establish score-based treatment strategies for UIAs.

## Introduction

Ruptured intracranial aneurysms are the most frequent cause of spontaneous subarachnoid hemorrhage (SAH), a type of hemorrhagic stroke (1, 2). Previously, brain aneurysms were typically diagnosed only after their rupture. However, the increasing use of cranial imaging has helped increase the detection of unruptured intracranial aneurysms (UIAs) (3, 4). While ruptured intracranial aneurysms necessarily require surgical or endovascular treatment (5–7), treatment decision making for UIAs is not straightforward because most UIAs remain asymptomatic, while a few eventually rupture leading to SAH (4, 8).

Based on a systematic review of prospective cohort studies with longitudinal follow-up of the course of UIAs, a risk prediction chart was developed, called the PHASES score (9). The PHASES score is based on six major predictors of UIA rupture (population, hypertension, age, size of aneurysm, earlier SAH, and site); it is used to predict the 5-year rupture risk of a UIA (9). More recently, a group of experts developed the Unruptured Intracranial Aneurysm Treatment Score (UIATS) (10). The UIATS considers the risk factors for aneurysm rupture and the clinical factors, which contribute to rupture risk or treatment risk. Based on these data, the score recommends either treatment or conservative management. In some cases, the UIATS can provide an inconclusive result, leaving the decision to the clinicians.

No large-scale prospective studies have assessed the score-based treatment strategies for patients with UIAs. Some clinical studies scored the aneurysms of patients with SAH and found that for many of the aneurysms that eventually ruptured, the scores indicated a low risk of rupture leading to the recommendation of no active treatment (11–15). Therefore, the authors argued that the scoring systems may not be robust enough to guide treatment decision making for UIAs. However, these studies analyzed only the ruptured aneurysms and did not include a control group, which was a major limitation of these studies.

We, therefore, set out to analyze the UIATS and PHASES score in a series of 40 consecutive SAH patients with *multiple* intracranial aneurysms. We aimed to evaluate how the PHASES score would estimate the rupture risk and which treatment recommendations UIATS would give for ruptured intracranial aneurysms and UIAs. We also examined whether the PHASES score and UIATS would discriminate between the ruptured aneurysms and the UIAs of a patient.

## Methods

### Ethics, Patients, and Data Collection

The study was approved by the Institutional Review Board (Ethikkommission der Landesärztekammer Rheinland-Pfalz) and was performed in accordance with the 1964 Helsinki declaration and its later amendments. Because the data were anonymized, and the study was retrospective, informed consent was waived.

We retrospectively identified all patients admitted to the Department of Neurosurgery of the University Medical Center of Mainz, Germany, between March 2010 and July 2016 with a diagnosis of spontaneous SAH (16, 17). From these, the patients diagnosed with multiple intracranial aneurysms were included in this study. In the cases with multiple aneurysms, the ruptured aneurysm was derived from the bleeding pattern.

All data were collected in anonymized tables. The data required for UIATS and PHASES score were extracted from the charts. To determine aneurysm characteristics, we analyzed the diagnostic images (computed tomography angiography and digital subtraction angiography) obtained on admission with Sectra software (Sectra Workstation IDS7, Version 17.1.10, Sectra AB, Linköping, Sweden). Tables 1A,B show the features collected to calculate UIATS and PHASES scores.

**Table 1A:** Features according to the Unruptured Intracranial Aneurysm Treatment Score (UIATS).

	**Factors**	**Levels**	**Repair**	**Conservative**	**RIA**	**UIA**
**Patient**	Age (single)	<40 years	4		6	10
		40–60 years	3		21	34
		61–70 years	2		8	11
		71–80 years	1		2	3
		>80 years	0		3	3
	Risk factor incidence (multiple)	Previous SAH from a different aneurysm	4		1	1
		Familial intracranial aneurysms or SAH	3		0	0
		Japanese, Finnish, Inuit ethnicity	2		0	0
		Current cigarette smoking	3		15	24
		Hypertension (systolic BP > 140 mm Hg)	2		22	32
		Autosomal-polycystic kidney disease	2		0	0
		Current drug abuse (cocaine, amphetamine)	2		3	5
		Current alcohol abuse	1		6	8
	Clinical symptoms related to UIA (multiple)	Cranial nerve deficit	4		0	0
		Clinical or radiological mass effect	4		0	0
		Thromboembolic events from the aneurysm	3		0	0
		Epilepsy	1		4	5
	Other (multiple)	Reduced quality of life due to fear of rupture	2		0	0
		Aneurysm multiplicity	1		40	61
	Life expectancy due to chronic and/or malignant	<5 years		4	6	9
	diseases (single)	5–10 years		3	0	0
		>10 years		1	34	52
	Comorbid disease (multiple)	Neurocognitive disorder		3	0	0
		Coagulopathies, thrombophilic diseases		2	2	3
		Psychiatric disorder		2	11	15
**Aneurysm**	Maximum diameter (single)	≤3.9 mm	0		7	44
		4.0–6.9 mm	1		20	14
		7.0–12.9 mm	2		12	3
		13.0–24.9 mm	3		1	0
		≥25 mm	4		0	0
	Morphology (multiple)	lrregularity or lobulation	3		10	5
		Size ratio >3 or aspect ratio >1.6	1		6	1
	Location (single)	Basal bifurcation	5		2	0
		Vertebral/basilar artery	4		3	7
		AcomA or PcomA	2		15	12
	Other (multiple)	Aneurysm growth on serial imaging	4		0	0
		Aneurysm *de novo* formation on serial imaging	3		1	0
		Contralateral stenoocclusive vessel disease	1		0	0
**Treatment**	Age-related risk (single)	<40 years		0	6	10
		40–60 years		1	21	34
		61–70 years		3	8	11
		71–80 years		4	2	3
		>80 years		5	3	3
	Aneurysm size-related risk (single)	<6 mm		0	17	57
		6.1–10 mm		1	19	3
		10.1–20 mm		3	4	1
		>20 mm		5	0	0
	Aneurysm complexity related risk (single)	High		3	11	9
		Low		0	29	52
	Intervention related risk (constant)			5	40	61

**Table 1B:** Factors according to PHASES score.

		**Points**	**RIA** **N**	**UIA** **N**
**P**opulation	North American/European	0	40	61
	Japanese	3	0	0
	Finnish	5	0	0
**H**ypertension	No	0	18	29
	Yes	1	22	32
**A**ge (years)	≤70	0	35	55
	>70	1	5	6
**E**arlier SAH	No	0	39	60
	Yes	1	1	1
**S**ite of aneurysm	ICA	0	6	14
	MCA	2	13	27
	ACA/Pcom/ACP	4	21	20
Aneurysm **S**ize (mm)	≤7.0	0	27	60
	7.1–9.9	3	9	0
	10–19.9	6	4	1
	≥20	10	0	0

### Calculation and Application of the Unruptured Intracranial Aneurysm Treatment Score and PHASES Score

The clinical characteristics were used to calculate the UIATS for each aneurysm (10). The subscore for conservative management was subtracted from the subscore for treatment to obtain the difference between the UIAT subscores (UIAT_DIFF_). A difference of more than two points, indicated a recommendation for treatment or conservative management. In cases with a difference of 0–2 points in either direction, the score was interpreted as inconclusive.

The PHASES score was calculated based on individual patient and aneurysm characteristics, as described (9).

### Statistics

Data analysis was performed using the SAS/STAT® software, version 9.4 of the SAS system for Windows (SAS Institute Inc., Cary, NC, USA) and R software version 4.0 (https://www.r-project.org/). Differences in PHASES score and UIATS between ruptured intracranial aneurysms and UIAs were determined by fitting linear mixed models with a patient-specific random intercept. The within-patient and between-patient standard deviations were estimated based on the fitted models. The regression coefficients were rescaled according to the within-patient standard deviations for comparing the ability of both scores to discriminate between ruptured aneurysms and UIAs of the same patient. Additionally, we computed areas under the receiver operating characteristic (ROC) curves for both scores for each patient's aneurysms and compared them statistically using the sign test. Conditional logistic regression models were fitted to the aneurysm type (ruptured aneurysm vs. UIA) to investigate the ability of the different aneurysm-specific features of the scores to discriminate between ruptured aneurysms and UIAs of the same patient. The likelihood ratio Chi-squared test and the Akaike information criterion (AIC) were used to compare the models, and revised scores were constructed based on the selected model coefficients. We then broke the one-to-many matching by patients and compared the pool of 40 ruptured aneurysms with that of 61 UIAs to calculate the sensitivity and specificity by applying the described cutoffs of the scores. We similarly generated ROC curves to analyze the ability of all scores to discriminate between ruptured intracranial aneurysms and UIAs, marginally. All *p*-values are two-sided. *P*-values below 0.05 were considered indicative of statistical significance.

## Results

### Patients and Characteristics of the Aneurysms

A total of 284 patients admitted between March 2010 and July 2016 to the Department of Neurosurgery of the University Medical Center of Mainz (Germany) with the diagnosis of spontaneous SAH were identified. Of these, 246 were diagnosed with a ruptured aneurysm as the bleeding source, and 40 (16.3%) were diagnosed with multiple aneurysms. Twenty-eight patients were diagnosed with two, eight patients with three, and four patients with four or more aneurysms. The most frequent patient level risk factors were current smoking (15 patients, 40%) and arterial hypertension (22 patients, 55%). Notably, compared with the UIAs, the ruptured intracranial aneurysms were larger and had higher aspect ratios.

Tables 1A,B show the clinical and imaging features required for calculation of the UIATS and PHASES score for ruptured intracranial aneurysms and UIAs. The clinical characteristics of the patient cohort are shown in Table 2.

**Table 2:** Demographic and clinical characteristics of the study population.

**Sex**	
Male	7 (17.5%)
Female	33 (82.5%)
**Age**	
≤40	6 (15%)
41–60	21 (52.5%)
61–70	8 (20%)
71–80	2 (5%)
≥81	3 (7.5%)
**Aneurysm location**	
Basal bifurcation	2 (2.0%)
Vertebral/basilar artery	10 (9.9%)
AcomA/PcomA/ACP	29 (28.7%)
ICA	20 (19.8%)
MCA	40 (39.6%)
**Aneurysm count**	
2	28 (70%)
3	8 (20%)
≥4	4 (10%)

### UIATS and PHASES Score in Ruptured Intracranial Aneurysms and UIAs

#### Higher UIAT_DIFF_ and PHASES Scores in Ruptured Intracranial Aneurysms

**Figure 1:**
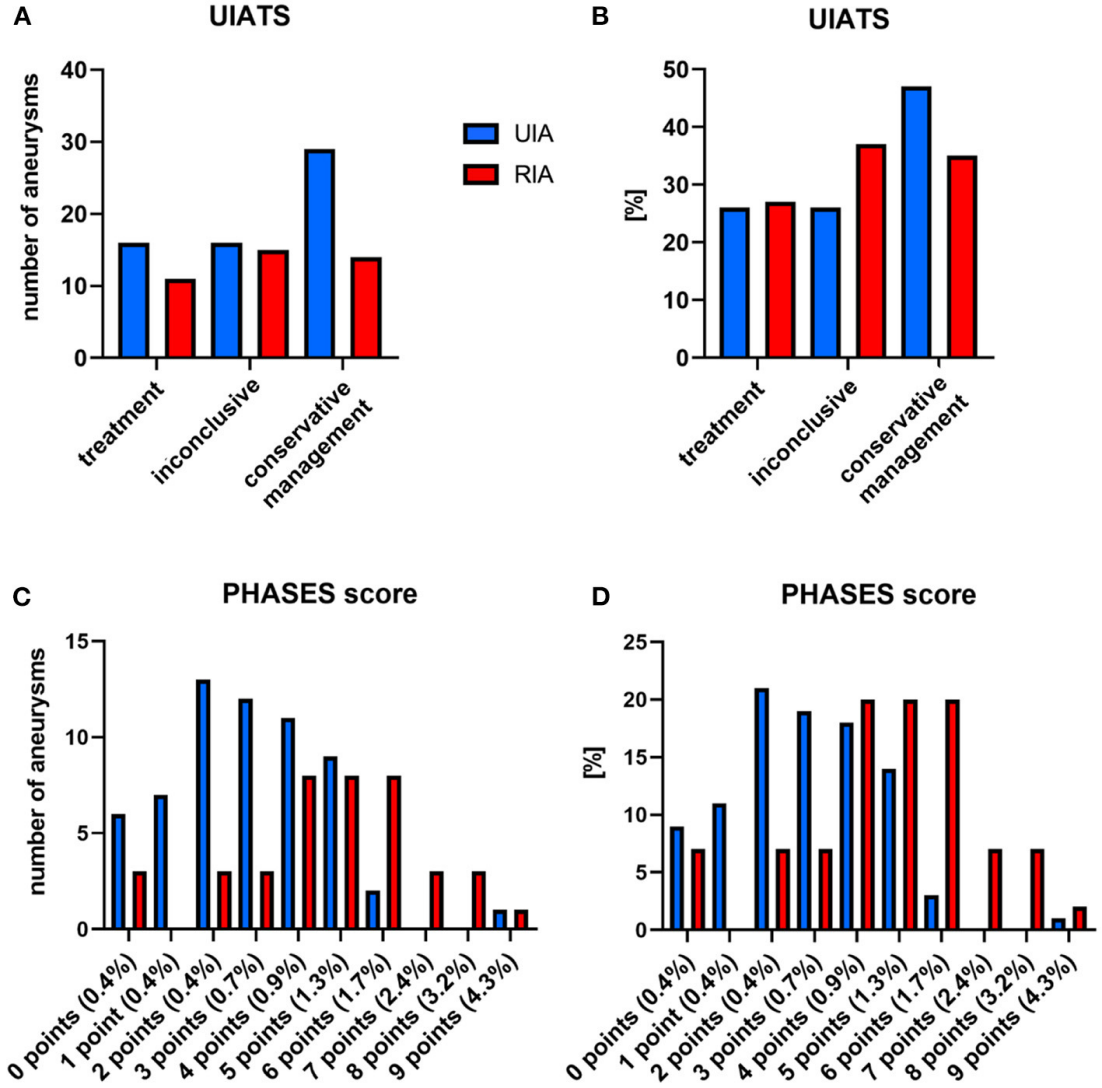
UIATS and PHASES scores in ruptured aneurysms (RIAs) and UIAs. **(A,B)** UIATS treatment recommendations for RIAs and UIAs in our cohort. **(C,D)** Five-year rupture risks of RIAs and UIAs in our cohort according to the PHASES score. **(A,C)** Absolute number of aneurysms; **(B,D)** percentage of UIAs/RIAs. UIA, unruptured intracranial aneurysm; RIA, ruptured intracranial aneurysm.

For the 40 ruptured aneurysms, UIATS recommended aneurysm treatment in 11 cases (27.5%), conservative management in 14 cases (35%), and was inconclusive in 15 cases (37.5%). For the 61 UIAs, UIATS recommended aneurysm treatment in 16 (26.2%), conservative management in 29 (47.5%), and was inconclusive in 16 (26.2%) cases. A detailed overview of the UIATS and the distribution of the PHASES score for ruptured aneurysms, and UIAs is shown in Figure 1.

In the ruptured intracranial aneurysms, the PHASES score and the difference between the UIAT subscores favoring treatment and favoring conservative management (UIAT_DIFF_) were higher compared with the UIAs in the same patient. Based on the linear mixed model analysis the adjusted means of the UIAT_DIFF_ (ruptured intracranial aneurysms vs. UIAs) were −0.60 (95% CI −2.11–0.91) vs. −1.45 (95% CI −2.80−0.11). The difference was 0.85 (95% CI −0.05–1.76, *p* = 0.064); for the PHASES score: 4.73 (95% CI 4.05–5.40) vs. 2.92 (95% CI 2.37–3.47). The difference was 1.80 (95% CI 0.96–2.65, *p* < 0.0001).

#### Discriminatory Power of UIATS and PHASES Score

The within-patient standard deviations of UIAT_DIFF_ and PHASES score in UIAs were 1.88 and 1.75, respectively. After rescaling each score accordingly, the standardized mean differences between ruptured aneurysms and UIAs were 0.45 (95% CI −0.03–0.94) for UIAT_DIFF_ and 1.03 (95% CI 0.55–1.51) for the PHASES score. This indicates that the PHASES score discriminated better than the UIAT_DIFF_ between the ruptured aneurysms and UIAs within patients.

The between-patient standard deviation was higher for UIATS than for the PHASES score (4.13 vs. 0.84). The resulting intraclass correlation coefficients were 0.83 (UIATS) and 0.19 (PHASES score). This clearly demonstrated the prominent role of patient-specific items that are part of the UIATS, which, conversely, can reasonably be expected to be helpful for treatment decisions. Unfortunately, our study design does not allow conclusions on this aspect.

**Figure 2:**
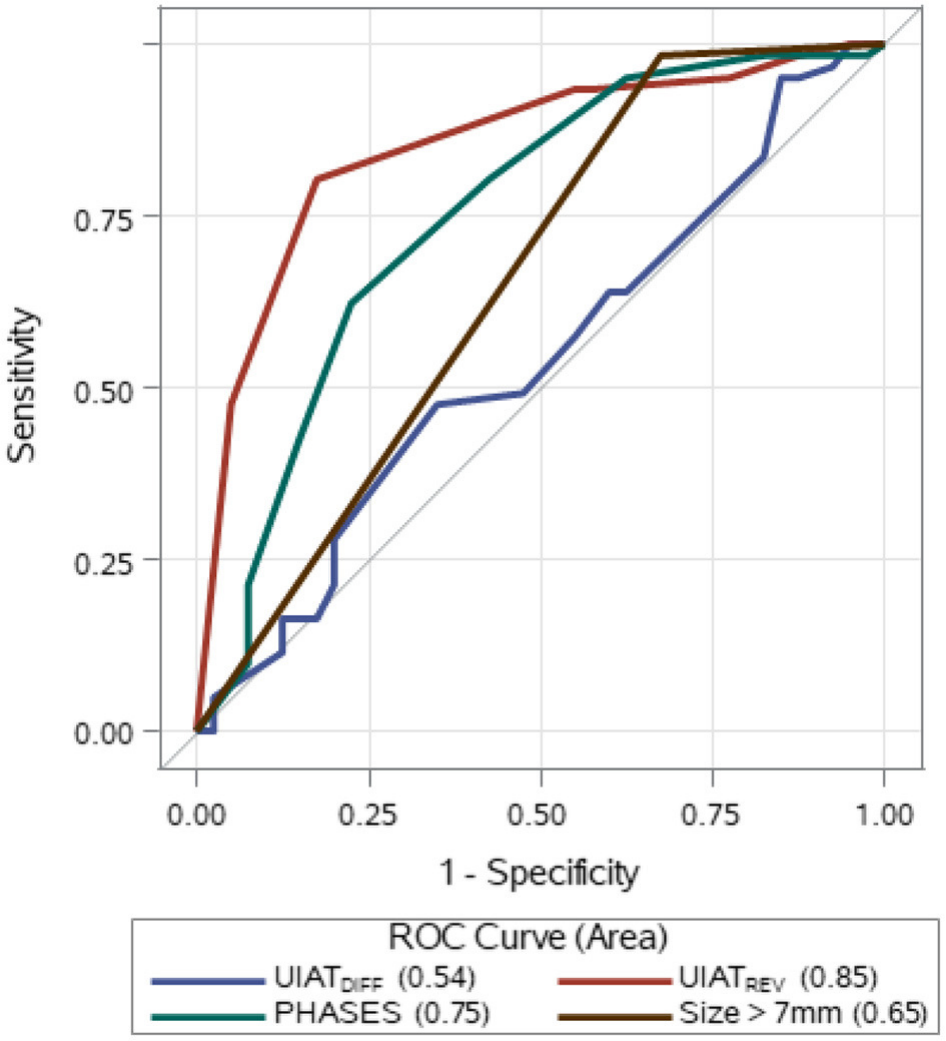
UIATS, PHASES score, aneurysm size, and revised score UIAT_REV_: ROC curve analysis. The AUC for UIAT_DIFF_ was 0.54 (95% CI 0.42–0.66), for PHASES score 0.75 (95% CI 0.65–0.85), for UIAT_REV_ 0.85 (95% CI 0.77–0.93), and for size 0.65 (95% CI 0.58–0.73). UIAT_DIFF_: difference between the UIAT subscores favoring treatment and favoring conservative management; ROC, receiver operating characteristic. Size > 7 mm: score according to the feature “size” of the PHASES score. UIAT_REV_, revised score based on features of UIATS: UIAT_REV_ = 2 × maximum diameter points + location points + 2 × size-related risk points.

To calculate sensitivity and specificity, the UIATS and PHASES score were applied to the ruptured aneurysms and the UIAs, assuming that for ruptured aneurysms, the decision for treatment, and for UIAs for conservative management would be correct. A PHASES score of ≥4 points was considered as a recommendation for treatment, as a score of ≤3 indicated a low likelihood of aneurysm rupture in another study (18). With these settings, UIATS recommended treatment in 11 of 40 ruptured aneurysms and 16 of 61 UIAs, resulting in a sensitivity of 28% and a specificity of 74%. The PHASES score recommended treatment in 31 of 40 ruptured aneurysms and in 23 of 61 UIAs, resulting in a sensitivity of 78% and a specificity of 62%. To investigate the scores' ability to discriminate between ruptured aneurysms and UIAs, a ROC curve analysis on PHASES score and UIAT_DIFF_ was performed; the results are shown in Figure 2. The areas under the ROC curves (AUC) for the PHASES score (0.75) was larger than that for UIAT_DIFF_ (0.54).

We also evaluated the ability of UIATS and PHASES score to discriminate between ruptured aneurysms and UIAs of the same patient. The average patient-specific AUC were 0.65 (SD 0.42) for the UIAT_DIFF_ and 0.74 (SD 0.35) for the PHASES score. The patient-specific AUCs were identical in 26 patients. PHASES score outperformed UIATS in eight patients and vice versa in six patients (*p* = 0.79, sign test).

Taken together, the findings indicate a higher discriminatory power between ruptured aneurysms and UIAs of the PHASES score compared with UIATS. However, the information provided by this evaluation is limited by the study design because prospective data on the course of the UIAs are not available.

#### Aneurysm Size Is the Most Robust Predictor of Rupture

By fitting conditional logistic regression models, we investigated whether the features of UIATS were able to predict the ruptured aneurysm within patients by using other weights or by discarding some features. We started entering the variable aneurysm-specific features. They performed better than UIAT_DIFF_ [*p* = 0.0001 (likelihood ratio Chi-squared test); AIC 50.8]. After stepwise elimination, a model with three features (maximum diameter, location, and size-related risk) did not perform worse (AIC 44.4). Using rounded regression coefficients, we constructed a simplified reweighted score (UIAT_REV_ = 2 × maximum diameter + location + 2 × size-related risk). Similarly, we investigated the features of the PHASES score. The binary variable size > 0 alone performed no worse than the PHASES score (likelihood ratio Chi-squared: PHASES 17.7 vs. 22.7 for the binary size variable, both *p* < 0.0001).

We calculated areas under the unconditioned ROC curve (Figure 2). UIAT_REV_ performed better than the PHASES score (AUC: 0.85 vs. 0.75). However, the single binary feature “size” extracted from the PHASES score performed worse than the unrevised PHASES score (AUC: 0.65 vs. 0.75). Collectively, these findings indicate that aneurysm size is the most robust predictor of rupture. However, aneurysm size is not optimally weighted and categorized in both scores to discriminate between ruptured aneurysms and UIAs in our setting.

## Discussion

In this study, we collected morphological information on aneurysms and the clinical characteristics of 40 consecutive patients who experienced an SAH from one of multiple intracranial aneurysms. The prevalence of multiple aneurysms in our cohort was 16.3% of all patients who experienced an aneurysmal SAH, which is within the expected range (19, 20). The clinical features were comparable with other SAH patient cohorts (1, 2). Although UIATS and PHASES scores were developed from prospective data on UIAs (9, 10), we applied the scores to our SAH cohort. We were therefore able to evaluate how the PHASES score would estimate the rupture risk and which treatment recommendations UIATS would give for the ruptured intracranial aneurysms and the UIAs.

In our cohort, the PHASES score and the difference between the UIATS subscores were higher in the ruptured intracranial aneurysms compared with the UIAs, which reached the level of significance for the PHASES score. The PHASES score estimated a low 5-year rupture risk in a relatively large proportion of the ruptured aneurysms (≤1.7% for 82.5% and ≤0.7% for 22.5%). This observation is similar to other studies on SAH cohorts, which found rather low PHASES scores in a large proportion of ruptured aneurysms (11–15). However, it should be noted that in our study, the PHASES score estimated a low 5-year rupture risk in a markedly larger proportion of the UIAs (≤0.7% in 62.3%, ≤1.7% in 98.4%). In a study conducted in a northwestern European population, UIAs were incidentally detected in 1.8% of 2,000 brain MRI scans (21). Furthermore, in studies evaluating aneurysms in SAH cohorts, there is a strong element of selection bias since only SAH patients are included. Taken together, these facts may partly explain the apparent divergence between the clinical observation that a large proportion of SAH patients have small ruptured aneurysms and their low predicted rupture risk.

Further analysis showed a higher power for the PHASES score compared with UIATS in discriminating between ruptured aneurysms and UIAs. This is most likely because aneurysm size, which we found to be the most robust discriminator between ruptured aneurysms and UIAs, has a greater influence on the PHASES score—the UIATS places a high weight on aneurysm size-related treatment risk, which is in favor of conservative treatment and evens out the points awarded for aneurysm size and in favor of treatment. In our data, a revised UIATS placing more weight on aneurysm size showed higher power to discriminate between ruptured aneurysms and UIAs. However, this finding needs to be validated in future studies. Based on the scores [UIATS recommendation and the PHASES score with a cutoff of ≥4 points (18)], a considerable proportion of the UIAs would have been treated. However, the seemingly high proportion of UIAs incorrectly deemed to be at high risk of rupture is not necessarily indicative of bad performance because this group of UIAs may include high-risk aneurysms. A key limitation of this study is the lack of longitudinal data or follow-up to show the subsequent rate of rupture of the unruptured aneurysms because the majority of the UIAs were subsequently treated.

Treatment recommendations should not be based solely upon the aneurysm rupture risk as the individual treatment risk should be considered to give the optimal personalized treatment recommendation. This aspect is not reflected by the PHASES score. In contrast, the UIATS, which was developed as a tool to aid clinical decision making, considers several factors that reflect the individual treatment risk and should presumably be included in the clinical decision process. Unfortunately, our study design does not allow conclusions on whether these treatment risk factors are helpful for clinical decision making.

## Conclusions

We applied UIATS and PHASES score to ruptured aneurysms and UIAs in an SAH patient cohort with multiple intracranial aneurysms. To the best of our knowledge, this is the first study to do so. Similar to previous studies on other SAH cohorts, a significant proportion of the ruptured aneurysms exhibited an apparently low-rupture risk, however, this phenomenon may be related to the selection bias in our SAH cohort and therefore may not be generalizable. Nevertheless, PHASES score discriminated between ruptured aneurysms and UIAs in our cohort. UIATS exhibited a lower discriminatory power, which was due to the high weight of aneurysm-independent factors. However, these patient-specific factors, presumably, are of high clinical significance to estimate the individual treatment risk. Altogether, our data support a careful acknowledgment of the scores in individual treatment decisions. Treatment decisions remain challenging in patients with UIAs, and prospective trials are warranted to evaluate treatment strategies based on UIATS and PHASES score.

## Data Availability Statement

The raw data supporting the conclusions of this article will be made available by the authors, without undue reservation.

## Ethics Statement

The studies involving human participants were reviewed and approved by Ethikkommission der Landesärztekammer Rheinland-Pfalz. Written informed consent for participation was not required for this study in accordance with the national legislation and the institutional requirements.

## Author Contributions

AN and SK conceptualized and designed the study. TP and AN acquired the data. AN, TP, and SK drafted the manuscript. AN, TP, JK, MB, FR, and SK performed critical revision of the manuscript for important intellectual content. AN, TP, JK, and SK analyzed and interpreted the data. All authors contributed to the article and approved the submitted version.

## Conflict of Interest

The authors declare that the research was conducted in the absence of any commercial or financial relationships that could be construed as a potential conflict of interest.

## References

[1] Lawton MT Vates GE. Subarachnoid hemorrhage. N Engl J Med. (2017) 377:257–66. 10.1056/NEJMcp1605827 28723321

[2] Macdonald RL Schweizer TA. Spontaneous subarachnoid haemorrhage. Lancet. (2017) 389:655–66. 10.1016/S0140-6736(16)30668-7 27637674

[3] Gabriel RA Kim H Sidney S McCulloch CE Singh V Johnston SC. Ten-year detection rate of brain arteriovenous malformations in a large, multiethnic, defined population. Stroke. (2010) 41:21–6. 10.1161/STROKEAHA.109.566018 19926839 PMC2847493

[4] Etminan N Rinkel GJ. Unruptured intracranial aneurysms: development, rupture and preventive management. Nat Rev Neurol. (2016) 12:699–713. 10.1038/nrneurol.2016.150 27808265

[5] Diringer MN Bleck TP Claude Hemphill J 3rd Menon D Shutter L Vespa P. Critical care management of patients following aneurysmal subarachnoid hemorrhage: recommendations from the Neurocritical Care Society's Multidisciplinary Consensus Conference. Neurocrit Care. (2011) 15:211–40. 10.1007/s12028-011-9605-9 21773873

[6] Connolly ES Rabinstein AA Jr Carhuapoma JR Derdeyn CP Dion J Higashida RT. Guidelines for the management of aneurysmal subarachnoid hemorrhage: a guideline for healthcare professionals from the American Heart Association/american Stroke Association. Stroke. (2012) 43:1711–37. 10.1161/STR.0b013e3182587839 22556195

[7] Steiner T Juvela S Unterberg A Jung C Forsting M Rinkel G. European Stroke Organization guidelines for the management of intracranial aneurysms and subarachnoid haemorrhage. Cerebrovasc Dis. (2013) 35:93–112. 10.1159/000346087 23406828

[8] Marbacher S Diepers M Kahles T Nedeltchev K Remonda L Fandino J. Interdisciplinary decision-making and treatment of intracranial aneurysms in the era of complementary microsurgical and endovascular techniques. Swiss Med Wkly. (2016) 146:w14372. 10.4414/smw.2016.14372 27878787

[9] Greving JP Wermer MJ Brown RD Morita A Jr Juvela S Yonekura M. Development of the PHASES score for prediction of risk of rupture of intracranial aneurysms: a pooled analysis of six prospective cohort studies. Lancet Neurol. (2014) 13:59–66. 10.1016/S1474-4422(13)70263-1 24290159

[10] Etminan N Brown RD Beseoglu K Jr Juvela S Raymond J Morita A. The unruptured intracranial aneurysm treatment score: a multidisciplinary consensus. Neurology. (2015) 85:881–9. 10.1212/WNL.0000000000001891 26276380 PMC4560059

[11] Foreman PM Hendrix P Harrigan MR Fisher WS. 3rd, Vyas NA, Lipsky RH, et al. PHASES score applied to a prospective cohort of aneurysmal subarachnoid hemorrhage patients. J Clin Neurosci. (2018) 53:69–73. 10.1016/j.jocn.2018.04.014 29685416

[12] Hilditch CA Brinjikji W Tsang AC Nicholson P Kostynskyy A Tymianski M. Application of PHASES and ELAPSS scores to ruptured cerebral aneurysms: how many would have been conservatively managed? J Neurol Sci. (2018) 65:33–7. 10.23736/S0390-5616.18.04498-3 29808636

[13] Neyazi B Sandalcioglu IE Maslehaty H. Evaluation of the risk of rupture of intracranial aneurysms in patients with aneurysmal subarachnoid hemorrhage according to the PHASES score. Neurosurg Rev. (2019) 42:489–92. 10.1007/s10143-018-0989-2 29948496

[14] Pagiola I Mihalea C Caroff J Ikka L Chalumeau V Iacobucci M. The PHASES score: to treat or not to treat? Retrospective evaluation of the risk of rupture of intracranial aneurysms in patients with aneurysmal subarachnoid hemorrhage. J Neuroradiol = *Journal de neuroradiologie*. (2020) 47:349–52. 10.1016/j.neurad.2019.06.003 31400432

[15] Stumpo V Latour K Trevisi G Valente I D'Arrigo S Mangiola A. Retrospective application of UIATS recommendations to a multicenter cohort of ruptured intracranial aneurysms: how it would have oriented the treatment choices? World Neurosurg. (2020) 147:e262–e271. 10.1016/j.wneu.2020.12.041 33326858

[16] Neulen A Pantel T Dieter A Kosterhon M Berres M Thal SC. Volumetric analysis of intracranial vessels: a novel tool for evaluation of cerebral vasospasm. Int J Comput Assist Radiol Surg. (2019) 14:157–67. 10.1007/s11548-018-1844-1 30097958

[17] Neulen A Kunzelmann S Kosterhon M Pantel T Stein M Berres M. Automated grading of cerebral vasospasm to standardize computed tomography angiography examinations after subarachnoid hemorrhage. Front Neurol. (2020) 11:13. 10.3389/fneur.2020.00013 32082241 PMC7002561

[18] Bijlenga P Gondar R Schilling S Morel S Hirsch S Cuony J. PHASES score for the management of intracranial aneurysm: a cross-sectional population-based retrospective study. Stroke. (2017) 48:2105–12. 10.1161/STROKEAHA.117.017391 28667020

[19] Wiebers DO Whisnant JP Huston J. 3rd, Meissner I, Brown RD, Piepgras Jr. DG, et al. Unruptured intracranial aneurysms: natural history, clinical outcome, and risks of surgical and endovascular treatment. Lancet. (2003) 362:103–10. 10.1016/S0140-6736(03)13860-3 12867109

[20] Morita A Kirino T Hashi K Aoki N Fukuhara S Hashimoto N. The natural course of unruptured cerebral aneurysms in a Japanese cohort. N Engl J Med. (2012) 366:2474–82. 10.1056/NEJMoa1113260 22738097

[21] Vernooij MW Ikram MA Tanghe HL Vincent AJ Hofman A Krestin GP. Incidental findings on brain MRI in the general population. N Engl J Med. (2007) 357:1821–8. 10.1056/NEJMoa070972 17978290

